# Staff Perception on Biomedical or Health Care Waste Management: A Qualitative Study in a Rural Tertiary Care Hospital in India

**DOI:** 10.1371/journal.pone.0128383

**Published:** 2015-05-29

**Authors:** Sudhir Chandra Joshi, Vishal Diwan, Ashok J. Tamhankar, Rita Joshi, Harshada Shah, Megha Sharma, Ashish Pathak, Ragini Macaden, Cecilia Stålsby Lundborg

**Affiliations:** 1 Department of Community Medicine, R.D. Gardi Medical College, Ujjain, India; 2 Department of Public Health and Environment, R.D. Gardi Medical College, Ujjain, India; 3 International Center for Health Research, R.D. Gardi Medical College, Ujjain, India; 4 Department of Public Health Sciences, Karolinska Institutet, Stockholm, Sweden; 5 Indian Initiative for Management of Antibiotic Resistance, Department of Environmental Medicine, R.D. Gardi Medical College, Ujjain, India; 6 Department of Microbiology, R.D. Gardi Medical College, Ujjain, India; 7 Department of Pharmacology, R.D. Gardi Medical College, Ujjain, India; 8 Department of Pediatrics, R.D. Gardi Medical College, Ujjain, India; 9 Department of Women and Children’s Health, International Maternal and Child Health Unit, Uppsala University, Uppsala, Sweden; 10 St. Johns Research Institute, Bangalore, India; London School of Hygiene and Tropical Medicine, UNITED KINGDOM

## Abstract

**Background:**

Health care or biomedical waste, if not managed properly, can be of high risk to the hospital staff, the patients, the community, public health and the environment, especially in low and middle income settings where proper disposal norms are often not followed. Our aim was to explore perceptions of staff of an Indian rural tertiary care teaching hospital on hospital waste management.

**Method:**

A qualitative study was conducted using 10 focus group discussions (FGDs), with different professional groups, cleaning staff, nurses, medical students, doctors and administrators. The FGD guide included the following topics: (i) role of Health Care Waste Management (HCWM) in prevention of health care associated infections, (ii) awareness of and views about HCWM-related guidelines/legislation, (iii) current HCWM practices, (iv) perception and preparedness related to improvements of the current practices, and (v) proper implementation of the available guidelines/legislation. The FGDs were recorded, transcribed verbatim, translated to English (when conducted in Hindi) and analysed using content analysis.

**Results:**

Two themes were identified: Theme (A), ‘Challenges in integration of HCWM in organizational practice,’ with the categories (I) Awareness and views about HCWM, (II) Organizational practices regarding HCWM, and (III) Challenges in Implementation of HCWM; and Theme (B), ‘Interventions to improve HCWM,’ with three categories, (I) Educational and motivational interventions, (II) Organizational culture change, and (III) Policy-related interventions.

**Conclusion:**

A gap between knowledge and actual practice regarding HCWM was highlighted in the perception of the hospital staff. The participants suggested organizational changes, training and monitoring to address this. The information generated is relevant not merely to the microsystem studied but to other institutions in similar settings.

## Introduction

Health care waste also termed biomedical waste contains infectious, contaminated and hazardous waste like discarded sharps, non-sharps, blood, body parts, toxic chemicals, pharmaceuticals, medical devices and radioactive substances [1]. If not managed properly, it carries a substantial risk to the hospital staff, the patients, the community, public health and environment.

The process of health care waste management (HCWM) involves challenging issues like collection and segregation, timely removal and safe disposal, illegal scavenging, patient safety, occupational safety and environmental safety [1–2]. Various steps in the process are mainly engineering functions, yet initial segregation and storage of HC-waste are the responsibilities of health care workers[3]**.** During the recent past, effective and efficient stepwise HCWM has emerged as a critical component in control of healthcare associated infections (HAIs). In high income countries, a combination of stringent application of legal provisions with other inputs has been effective in mitigating the menace of health-care waste [4]**.**

Among the limited number of studies available from resource poor nations in this context, some have informed regarding the deficiencies of HCWM in these countries and highlighted the reasons as to why HCWM in these countries is inadequate and fraught with difficulty. These reasons include—want of stringent application of rules, lack of adequate knowledge, awareness and motivation, dearth of appropriate technological interventions, improper management-strategies, inadequate funds or a mix of all [1,5–15]. Among these, some studies are interview and observation based [7, 12, 13,15].

In India, “Bio Medical Waste Management and Handling Rules” were framed in 1998 [16]. Since then the onus lies on the health care institutions to ensure proper HCWM [3].However, much is unknown regarding the compliance of the norms [4]. In general, and specifically for the Indian context, there is a considerable paucity of management-strategy related research and context specific explorations of hospital staff perception regarding feasibility and acceptability of HCWM interventions and implementation strategies, particularly in rural hospital settings [17–19].

In view of the foregoing, we performed a qualitative study in a rural tertiary care teaching hospital in India to explore the perceptions of the various professional groups working in the hospital regarding health care waste management

## Methods

The study was conducted in a 570-bed rural tertiary care teaching hospital in Ujjain, India, from June 2009 to January 2010, among 75 staff-members belonging to five professional groups—cleaning staff, nurses, medical students, doctors and administrators (Table 1). Ethical approval (dnr 169/2011) was granted by the ethics committee of the R.D. Gardi Medical College, Ujjain.

**Table 1:** Characteristics of participants in focus group discussions on health care waste management in a rural hospital in India.

FGD Number	Group and Category	Number of Participants			Age-range (years)	Qualification (number)
		Total	Male	Female		
1	Medical students	6	4	2	22–23	Medical students (6)
2	Doctors	7	5	2	26–63	PG Doctors (5), Graduate Doctor. (1), M.Sc., Ph.D.(Microbiology) (1)
3	Nurses	8	-	8	20–25	10^th^ (1), 12^th^ (3), Science graduate and DMLT (1) Arts PG (2), GNM(1)
4	Nurses	6	-	6	23–34	10^th^ (1), 11^th^ (1), 12^th^ (1), Science graduate (1), Arts PG (2)
5	Medical students	8	5	3	20–22	Medical students (8)
6	Cleaning staff	8	4	4	18–48	10^th^ (2), 8^th^ (1), 5^th^ (1), Illiterate (4)
7	Cleaning staff	9	4	5	18–45	12^th^ (1), 10^th^(1), 9^th^(1), 8^th^(1), 5^th^ (1), Illiterate (4)
8	Senior administrators	6	4	2	52–68	PG Doctors (4), Graduate Doctor (1) Nursing In charge (1)
9	Doctors	8	6	2	28–61	PG Doctors (7), Graduate Doctors (1)
10	Nursing administrators	9	4	5	22–42	Science Graduate (2), Arts Graduate (1), GNM (3), ANM (3)
Total		75	36	39		

Note: PG—post graduate; Ph.D.—Doctor of Philosophy; DMLT—Diploma in Medical Laboratory Technique; B.Sc.- Bachelor of Science; B.A.—Bachelor of Arts; GNM—General Nursing & Midwifery; ANM—Auxiliary Nurse Midwife; Numbers such as 12^th^,11^th^,10^th^,9^th^,8^th^, 5^th^, indicate numbers of years of schooling.

### Data collection

The data was collected in conjunction with the data collection for a previous paper, where details of participants, data collection and data analyses have been presented [20]. In short, ten focus group discussions (FGD) were conducted, two for each professional group (Table 1). FGDs were chosen to get the benefit of interaction between different members of a group. To separate FGDs by profession was considered important to facilitate a free and open discussion among the participants within each group, the setting being traditional and hierarchical, where staff in lower positions do not express freely in the presence of staff of higher position.The FGDs were conducted in English or Hindi by SCJ, RJ, HS,MS and VD, one or two of them acting as facilitators in rotation and the others as observer/note-taker. The FGD guide was developed in several steps. After a review of the available literature and observations in the hospital, the authors developed a preliminary topic guide that was tested in two pilot FGDs. Discussions in the debriefing sessions after these FGDs among the four above mentioned researchers led to development of a draft FGD guide which was then discussed by the entire research group and a final FGD guide was developed. The final FGD guide included the topics: (i) role of HCWM in prevention of HAIs, (ii) awareness and views regarding HCWM related guidelines/legislation, (iii) current HCWM practice (iv) views on improvements in the current practices and (v) proper implementation of available guidelines/legislation. Each FGDs was conducted until saturation was reached and no further new information wasforthcoming. Members checks and researcher triangulation was used to validate the findings. Each FGD was recorded, transcribed and translated to English when conducted in Hindi. The participants were given prior information about the subject of the FGDs and their written consent was obtained. Participation was voluntary.

### Data analysis

Manifest and latent content analysis was used for analysing the transcripts [21]. Meaning units were extracted from the transcripts. A meaning unit is part of the original transcript that carries a specific meaning, and generally consists of several words taken directly from the transcript. Themeaning units were then condensed to condensed meaning units and further condensed into codes. Similar codes were grouped into sub-categories and further grouped into categories and ultimately the underlying themeswere identified [20,21]. Two co-authors performed the coding independently. No pre-defined coding scheme was used. The analysis was performed manually.

The results were repeatedly discussed among the authors who have varied backgrounds such as community medicine, clinical medicine, environmental medicine, medical microbiology, pharmacy and public health, have different genders and come from India and Sweden.

## Results

Characteristics of participants are presented in Table 1. Two themes were identified: (A)‘Challenges in integration of HCWM in organizational practice’(B) ‘Interventions to improve HCWM’. In the following account these themes are presented along with the categories and relevant quotations. Sub-categories and codes are shown in Tables 2 and 3. At appropriate places a description of the current practice and how it deviates from what is considered good practice is also given and threaded in the flow of the text. Explanations by the authors in quotations are added in square brackets.

**Table 2:** Theme A: Challenges in integration of HCWM in organizational practice.

Categories	Subcategories	Codes	Condensed meaning units
**Category I** Awareness and views about HCWM	a.Transmission of Health care associated infections (HAIs)	Resource constraints transmit HAIs	Diseases transmitted through health-care- waste
		HAIs via contaminated reusable objects	HAIs via other modes of transmission
	
	b.Prevention of HAIs	Hygiene in a holistic sense is very important	Two most important methods: HCWM and HH (Hand Hygiene)
		HAI prevention methods all equally important	Just proper disposal of infected material
	
	c.Hazards of improper Health care waste management (HCWM)	HAIs are transmitted in and through the hospital	New diseases can emerge
		Injury and infection by sharp objects	Fading of standard practices
	
	d. Awareness and thinking regarding stepwise process of proper HCWM	Awareness and thinking regarding collection of waste	Awareness and thinking regarding removal, transport and disposal of waste
**Category II** Organizational practices regarding HCWM	a. Organizational culture and current practices (OCCP) related to the stepwise process of proper HCWM	Waste-segregation and color coding	Filling limit of waste bins and bags
		Reuse/Disposal of used (contaminated) instruments	Littering and scattering of waste
		Accumulation of waste	Delay in removal and transport
		Open cart transportation of waste	Sorting of waste
		Resale of waste	Various techniques of waste transport and disposal
		Clean looking waste thrown behind hospital building	Open burning of waste in hospital backyard
		Incinerator	Waste disposal at small places
	
	b. OCCP related to protection from occupational hazards	Protection from accidental pricks and cuts	Demand for Personal Protective Equipment (PPE)
		Demand for Hepatitis B Vaccine	Demand for disinfectant and soap
**Category III** Challenges in Implementation of HCWM.	a. Reasons for non-adherence to guidelines	Lack of awareness and self discipline Lack of sense of hygiene	Lack of emphasis and strict authority
		Lack of imposition / motivation of discipline	Lack of facilities
		Lack of awareness and self discipline Lack of sense of hygiene	Lack of emphasis and strict authority
	
	b. Day to day problems in stepwise process of proper HCWM	Resource constraints	Logistic problems and language barrier
	

**Table 3:** Theme B: Interventions to improve HCWM.

Categories	Subcategories	Codes	Condensed meaning units
**Category I** Educational and motivational interventions	a.Enhancing awareness among the staff and general public	Application of mass media, and posters	On-the-job-instructions in the health-care-institutions
		Cultural programs in the fairs and festivals	Curricular and extra-curricular educational activities in the schools.
		Patients’ and their relatives’ and visitors’ education	
	b.Using reminders and reminding devices	Significance of reminders	Acceptability of reminders
		Category specific ways of reminding	Reminding devices
	Training	Relevance of training	Training only once versus continuous training
		Contents and frequency of training	Seminars
	c. Motivation-inputs	Performance feedback and positive reinforcement	Two way communication
		Competitions and prizes	Motivation during the routine rounds
**Category II** Organizational Culture Change	a.Providing more effective leadership	Infection Control Team (ICT) and Hospital Infection Control Committee (HICC)	Involvement of the staff through various roles and responsibilities
		Influence of the role-models	
	b.Proper response to needs and demands	Provision of proper infrastructure and facilities	Fulfillment of various needs and demands
**Category III** Policy-related interventions	a.Rules, regulations and Implementation	Rules and regulations needed	Implementation needed after framing the rules and regulations
		Forceful implementation	Rules should be combined with awareness and self discipline
	b.Supervision and monitoring	Supervisors needed	Supervisors not needed
		Responsibility of a supervisor	Power and authority of supervisors
		Monitoring;	Issuing ‘spot memo’
	c.Other policy related interventions	Contract to an agency	Appointing sanitary inspectors
		Type of employment: Daily wages or permanent	

### Theme A: ‘Challenges in integration of HCWM in organizational practice’

This theme emerged from three categories: (I) Awareness and views about HCWM, (II) Organizational practices regarding HCWM, and (III) Challenges in Implementation of HCWM.

#### Category I: Awareness and views about HCWM

In general, the staff was aware of the generation of the health care waste of various categories in the hospital like human anatomical waste, waste sharps, pharmaceutical waste, blood and body fluids waste, infectious waste. The awareness about it and the importance of it in the context of HCWM generally increased as their position in the official hierarchy increased from cleaning staff to nurses to chief administrator or professor or head of a department.

The hospital staff generally connected health care waste with the transmission of HAIs. Enumerating a good number of diseases (HIV, jaundice (hepatitis), diarrhoea, TB etc) transmitted through health-care waste, the cleaning staff said,

*“This filth can lead to several diseases”* Cleaning staff, FGD 6

Improper handling and disposal of waste at its origin was also pointed out.

The medical students emphasized, *“*....*the syringe should not be used again and again (to avoid transmission of diseases)”* Medical student, FGD 5

*“Improper disposal of bandages*, *syringes also spreads infections like HIV and hepatitis”*Medical Student, FGD 5

The hospital staff was aware of the shortcomings in the management of health care waste in the hospital and spoke about intermittent ‘littering and scattering of health-care waste in hospital premises’, instead of aggregating them at predefined storage places in a segregated form.They also disliked ‘delay in removal and transportation of the waste’,when not done in 24 hours (as recommended). They disapproved of‘open-cart transportation in the hospital with spillage around’, instead of in closed containers and transportation in open manner in the only elevator available in the hospital at any time during day,instead of at an ‘informed in advance specified time’. They also wanted disinfection of the elevator afterwards…

*“We [are supposed to]dispose sharp objects and needles etc*. *in a bucket but many of us throw away these on the floor*.*”* Cleaning staff, FGD 6

*“Today the situation is that the garbage remains for three continuous days in the same position*. *It is source of infection and it [removal] depends on the mercy of the hospital van that has been arranged for transportation of the waste*.*”* Senior administrator, FGD 8

*“What happens when the waste is not covered*, *the same goes in the same lift*. *The doctors are going in the same lift*, *patients are going in the same lift and they are taking it [waste] open*. *When we go on rounds at nine a*.*m*. *that waste transport man comes with his cart and all smell and foul smell and it is all open*, *these all pads and what not it is*, *that may be the cause of source of infection also*.*” Doctor*, *FGD 9*

All professional categories were of the opinion that segregation at source, which is the recommended practice, should be followed strictly and that the current practice of first mixing and then sorting the waste should be totally disallowed. All participants agreed about the importance of timely removal of waste in such a manner that segregation can be maintained until the terminal point:

*“Those doing the sorting [of mixed waste in large quantities]cleaning staff*, *nurses*, *medical students*, *doctors and administrators can get injured/infected*, *fall sick and the disease might spread to others*,.....*Hence it is better to practice the process of segregation at source*.*”* Cleaning staff, FGD 6

The discussions on management of HCW suggested that awareness did exist among the staff in higher positions like doctors and senior administrators. However, among the staff in lower positions, like cleaning staff there was absence of clarity about recommended practices especially regarding segregation of waste and the colour coded containers used for it. Some remarks presented here indicate their level of awareness.

*“All the waste can be disposed in a single bin except the sharp objects*.*”* Cleaning staff, FGD 6

*“Soiled things and filth are disposed in the black bucket*. *Water[liquid] is kept in the red bucket…similar colour-code for each ward*. .....*Blue coloured drums are there*. *Perhaps blue should be red*.*”* Cleaning staff, FGD 6

*“In order to sustain proper segregation*, *colour coded bags must be provided with the bins of respective colours; segregation is maintained better in this way*. *Yes*, *that is the gold standard*.*”* Senior administrator, FGD 8

‘Appropriate HCWM’ and Hand Hygiene (HH) was suggested as the two most important methods of HAI prevention.

“*Proper waste-disposal and hand washing are the most important methods for preventing hospital infection*.*”* Medical Student, FGD 1

#### Category II: Organizational practices regarding HCWM

Participants perceived that absence of work protocols and regulatory committees like Infection Control Team (ICT)/Hospital Infection Control Committee (HICC), remixing of segregated waste by the cleaning staff during collection/removal, lack of clarity about colour-codes,absence of colour-coded bags in the respective bins, not providing Personal Protective Equipment and inadequate supply of disinfectants/soap indicated deficiencies in the organization`s attitude towards HCWM. Thus they perceived these practices as important contributors to improper HCWM in the hospital. Participants, especially nursing and cleaning-staff particularly, discussed various issues related to the protection from accidental needle-stick injuries, proper disposal of sharps, absence of needle-cutters and the need of protocols for precautions before and after giving injections. The nursing staff informed about the absence or inadequacy of Hepatitis-B vaccine,while the cleaning staff’ informed about absence of Personal Protective Equipment and inadequate supply of disinfectant and soap. A member of cleaning staff summarised it succinctly,

*“Women in the cleaning staff are responsible for removal of the waste; they are doing it with bare hands and without washing their hands*. *The chances of developing diseases are high*. *They are at high-risk*. *We go home in the infected clothing and our children get infected when they come to us*. *If you want to protect us from the diseases then give us a special dress to wear*, *with gloves*, *and masks*.*”* Cleaning staff, FGD 7

#### Category III: Challenges in implementation of HCWM

In the opinion of the participants, the constraints for effective HCWM in the hospital were; inadequate resources, logistic problems, language barrier in training efforts (India being a multi-lingual society), and lack of sense of hygiene among the patients as well as among some individuals of the hospital staff. In addition, administrative will to change, training, monitoring, providing proper infrastructure and facilities were expressed as important to bring about the required improvements in HCWM.

*“We get the cleaning helpers with difficulty and frequently they are removed or replaced without intimating us*. ....... *There is shortage of staff*.*”*Nursing administrator, FGD10

*“The dustbins for segregation are not available*, *though general dustbins are there*.*Duty timings are so fixed*, *that in the morning many staff members are available*, *whereas in the night nobody*.*”* Nursing administrator, FGD 10

The administrators discussed implementation problems in greater details and one of them described many problems in one breath:

*“There is shortage of staff*. ........ *Everything is not possible for the brother or the sisters [nurses] to complete*. *So we need helping hands like aayabai*, *[female attendant-hands] or ward boy*, *at least sweeper and it is surprising that in the night also no sweeper*, *nobody is there*, *so that’s why all load is coming on cleaning staff which is coming in the morning*, *so it is quite difficult*. *Separate dustbins [colour coded bins for segregation] are also not available*. *…*.*say only general dustbin is there*.*And sometimes time water is not coming[to tap]*, *then it is more terrible and how they will wash their hands after touching an infected case or infected things*?*[if there is no water]*.*”* Nursing administrator, Nursing administrator, FGD 10

Senior administrators were especially concerned with issues such as hiring, contract and transport arrangements for health care waste disposal. They debated about the available service and alternatives in these areas. According to a senior administrator shortage of staff is there, but besides attitude of the cleaning staff is also an impediment,

*“A cleaning person would join*, *having worked for three days only*, *would run away telling it is hard work and too much of work load*. ....... *In fact the man he finds as a co-worker who had joined earlier*, *would say if you will work sincerely then everyone will have to do so*.*”* Senior administrator, FGD 8

### Theme B: “Interventions to improve HCWM”

Theme B emerged from three categories, (I) Educational and motivational interventions, (II) Organizational culture change, and (III) Policy-related interventions.

#### Category I: Educational and motivational interventions

Participants considered enhancing awareness among the staff and general public about proper HCWM as a prerequisite to all other interventions. In this context, influences of culture, schooling, family background, on-the-job-instructions and various reminding devices were discussed. Various methods perceived as relevant, effective and feasible included; appropriate use of mass media, banners and posters, training programs, cultural programs in fairs and festivals, and introducing relevant-curricular as well as extra-curricular inputs in the education system. Patients’ and their visitor`s education was also perceived as needed. Participants generally agreed that enhancing awareness will be preferable to enforcement:

*“Awareness is the basic thing which may eliminate the need of enforcement*.*”* Doctor, FGD 9

*“If people are aware*, *they can complain to the authorities and so such infections [as HAIs] can be prevented*.*”* Medical Student, FGD 1

Using illustrative/attractive posters was found to be everybody’s favourite method for both the processes—awareness generation as well as reminding.

*“Nurses cannot remind senior doctors in person; through written display/posters*, *they can get reminded [themselves]*.*”* Nurse, FGD 3

Seminars and training on proper HCWM were frequently suggested as special training-inputs. Training/refresher courses were perceived as an attractive intervention by all professional categories:

*“First training should be given then the [implementation of]rulescomes in picture*.*”* Medical Student, FGD 5

*“If training is not imparted we may continue committing several mistakes*.*”* Nurse, FGD 3

*“*....*we all clinicians are very enthusiastic for segregation of health care waste*, *but when sweeper comes*, *he pours all waste into one bucket(because of lack of training)*.*”* Doctor, FGD 9

The suggested interventions aimed at motivating the staff, included performance feedback and positive reinforcement, two way communication, competitions and prizes. Senior administrators and doctors suggested that regular efforts are to be made to motivate the doctors during the routine ward-rounds.

*“Training is required for all levels*.*”* Senior administrator, FGD 8

#### Category II: Organizational culture change

Participants stated that lack of facilities was the main problem in translating their willingness to comply with HCWM into actual practice. Provision of proper infrastructure and material emerged as a repeatedly discussed intervention in all the FGDs.

*“New unused gloves should be provided to us*. *There must be a bucket underneath every bed*. *If this facility is there then the waste will not be spread all over*.” Cleaning staff, FGD 6

Participants also stated that the important organizational culture changes needed were formulation of proper protocols; formation of Infection Control Team (ICT) and Hospital Infection Control Committee (HICC); delegation of responsibility and authority to the ICT, HICC, Heads of the Departments and Head Supervisors, who should also serve as role-models.

Generally the senior staff like doctors and senior administrators expressed that the ICT and HICC are cornerstones. The role of the staff is pivotal in executing the tenets of the HICC.

*“…*.*HICC forms cornerstone of everything*…*”* Doctor, FGD 9

#### Category III: Policy related interventions

Budget-provisions and regulatory force were much emphasised among many policy and implementation related suggestions. Some participants were in favour of forceful implementation of the framed rules whereas others preferred self-discipline cultivated through awareness drives:

*“Strict rules are needed and forceful implementation should be there …*.*from top to bottom*. *After publicity by hoardings[banners] etc*. *if they are not following the rules*, *then enforce the rules*.*”* Medical Student, FGD 5

A good number of participants were in favour of delegation of power and authority to supervisors coupled with regular monitoring:

*“Monitoring can be done by surprise checking*, *regular inspections*, *through daily or weekly rounds*, *with the help of photography*.*”* Nurse, FGD 3

*“Every week there should be one day when there should be a round from top to bottom including operation theatre*. *The culprits should be served notices*.*”* Senior Administrator, FGD 8

In addition, ‘contract to an agency’, appointing a specially trained manager’ or a ‘sanitary officer’ and recruiting special cleaning staff was also suggested.

*“After the training and awareness*, *somebody will have to take up the cudgels to implement and implement it at any cost*. *An administrator with adequate power is needed for hospital hygiene*.*”* Doctor, FGD 9

## Discussion

To our knowledge, this is the first FGD based qualitative study from India on health care waste management in which several professional categories of the hospital staff including the cleaning staff participated. The two themes that emerged (A) *‘Challenges in integration of HCWM in organizational practice’*(B) *‘Interventions to improve HCWM’*and the categories therein indicate the thought processes of the participants towards HCWM.

The participants’ general knowledge and awareness about various steps involved in the process of HCWM, hazards associated with health-care waste and prevention and transmission of HAIs was found according to their professional qualifications and position in the hierarchy.

Their preparedness to comply with the tenets of HCWM was also evident. However, among all professional groups, clarity regarding finer details of the practical aspects was lacking which appears to be due to absence of a system of proper HCWM practices in the hospital. This was due to deficiencies of infrastructure and facilities along with absence of an ongoing training and official-instructional activity related to the good practices of HCWM. For example, clarity about colour coding and practical- aspect-training in segregation and maintaining of HC-waste were unsatisfactory and this was found even among the senior administrators. The present regulation in India regarding HCWM practices came into force in 2011 i.e. after the data collection for this study [22]. One of the reasons for the update of the earlier regulation from 1998 was the confusion in the earlier regulation regarding which types of waste should be kept in which colour coded bag. This could thus be the reason behind some of the remarks made by our participants. A summary of the recommendations from the 1998 and 2011 regulations are given in S1 Appendix and S2 Appendix respectively[16, 22]

The major part of health care waste is made up of non-infectious and non hazardous waste which becomes infectious and hazardous when the two types get mixed. As per guidelines, segregation should be a rule without exception [22]. However in our setting we have observed and also it was highlighted in FGDs that some staff members follow proper segregation while others do not. As a result almost entire HCW is rendered infectious and hazardous. Further, waste is disposed anywhere leading to littering and scattering of the waste and also in wrong coloured bins marring segregation. For example, a syringe and needle after injection to an infectious patient should be disposed in the infectious waste bin, but instead it will be put in general waste bin and it will make infectious the whole non-infectious waste.

Other HCWM practices, besides in relation to colour coding, deviated however from the recommended HCWM practices at the time of the study. Statements of cleaning staff about prevalent practices of the doctors and nurses in general and those of nurses about senior doctors in particular portray a dismal scenario. The doctors claimed that they had been enthusiastic about HCWM. Several implementation problems were however highlighted by the administrators and doctors. These included lack of facilities, which was further emphasized by the nurses, cleaning staff and medical students. Earlier studies from India have similarly reported several deficiencies in implementation of guidelines and regulations of HCWM [17,23].

The findings of the present study further indicate a need to improve involvement, knowledge, skills and capabilities of the hospital staff making it a part of organizational-culture-change. For this a multipronged strategy is needed with an emphasis on providing better facilities and training. Several interventions, for example posters, reminders and role-models, which had been suggested for improving hand hygiene, were also suggested for waste management [20].

For a very long period, posters have been used to change attitudes and behaviour in health related matters. Attitudes can be changed using posters, but behaviour changes are more difficult [24,25]. According to protectiom motivation theory people will pay attention to those health threats that may put themsleves at risk. Further, in order to change behaviour it is important to indicate in a message what benefit people will gain by changing their behaviour [26–27]. While designing posters aimed at behavioural change it is helpful to consider theories or models of behaviour change and remember that people in different stages of change are susceptible to different behaviour modification strategies [28]. Therefore, the posters should also attempt to give graded messages suiting to varying audiances. Although posters may be effective in changing the behaviour for a short term, the efficacy of posters in terms of changing long term behaviour has not been established, Compared to posters ‘role-models’, particularly if they are local senior administrators, by their very nature, are persistent, live, interactive, persuasive and confidence inducive to help effect a long term behavioural change.

The issue of institutionalizing a powerful and accountable HICC bestowed with a mandate to apply rules and regulations stringently also came up during the discussions in the present study. Importance of authority to HICC has similarly been highlighted by a study from China [29].

Participants also suggested that the supervisors of the cleaning staff should administer among their staff, day to day reminders and educational inputs. For planning and administration of such interventions provision of separate healthcare-associated-infection-control-department and additional posts were suggested. This is in agreement with a study from Zimbabwe that recommended that the Infection Control Team should comprise of two nurses i.e. the infection control nurse should be supported by a matron [30].

Participants in the present study complained about poor or no adherence to waste-segregation at source. Unsatisfactory or even no segregation has been reported from a range of contexts earlier [31–35].Illegal resale and reuse of HC-waste has also been reported in several studies[2, 36, 37]. The participants in the present study perceived that sorting of HC-waste by cleaning staff for picking up of profitable items for resale and reuse could result in spread of infections. Participants also complained about delay in removal and disposal of waste. Participants pointed that this lapse can be responsible for transmission of infections through insect vectors and various vermin. According to one study this can take epidemic proportions [29].Intra-hospital transportation in open carts was an issue raised by the participants. Another Indian study too highlighted the need to address this problem suggesting that waste collection should be performed with aesthetic sense and the process should be completely free from the possibility of spillage [3].

In our study we found that senior level functionaries who themselves are responsible for providing essential inputs such as training and facilities do not seem to find fault with their own inability to provide these, but instead show a tendency to find fault with the juniors. Likewise the juniors seem to find fault with the senior management's inability to provide sufficient supplies. Such tendencies are explained as the ‘the theory of attribution’ wherein, for a happening, involved parties put the blame on others, but do not accept the blame themselves [38].

At the macroscopic level, factors effective in success of HCWM appear to be, financial and policy support on one hand and willingness and regulatory push on the other hand. Notwithstanding the fact that the present study is limited to a single micro-system, the interventions suggested in this study shown in Table 3, can be viewed in the light of above mentioned avenues of success. By doing so it emerges that the interventions belonging to category III (akin to regulatory push and financial and policy supports) and category II (akin to willingness) are essential for implementation of those belonging to category I. Since similar situations are prevailing in many parts of the world, these findings are relevant in developing better understanding of the issues and to develop suitable interventions. In a recent paper from China with similar contextualities, participants from a range of hospitals in a questionnaire study suggested interventions that were in agreement with what was suggested by our participants [39].

This study provides an insight into what the hospital staff perceives about HCWM and their current practices, how do they act and react, what are their training and other needs, what is immediately possible and acceptable and what is not. The study found evidence of a gap between theoretical thinking and actual practice. Awareness and preparedness were satisfactory whereas clarity, updating, involvement, emphasis, basic provisions and overall management appeared lacking. As the study explores perception of hospital staff about the HCWM process right from generation of HC waste to its removal from the hospital, the generated information will be helpful in developing appropriate management strategies for improving HCWM in many similar settings.

### Methodological considerations

FGDs were conducted with various groups of health providers from administrators to cleaning staff to bring out diverse perception. FGDs of health providers were conducted group-wise according to their roles and designations to facilitate free discussion as in a hierarchical society like India juniors do not express freely in the presence of seniors. With authors of different educational and country background, various perspectives have got included in the study. We conducted this study in a rural hospital of central India, however the findings might well be transferable to other hospitals with similar settings and infrastructure. Moderators for this study were from same medical college from where participates were recruited. It is possible that some participants may not have expressed their views completely freely. On the other hand, that the participants knew the moderators might have led to a more open and free discussion.

## Conclusion

The study found evidence of a gap between knowledge and actual practice, the so called know-do gap. The general awareness about procedures of HCWM and preparedness for involvement in improvement of current practices was evident. However, clarity about procedures of HCWM was not adequate among some of the study participants. The participants perceived that at the organizational level, there were deficiencies in offering basic provisions and a lack of emphasis by the administration on improving the HCWM. Participating staff suggested various interventions like organizational changes, greater involvement of the staff, regular training courses, continuous monitoring, stringent rules and regulations and incentives and disincentives to address these deficiencies. The information generated is relevant not merely to the microsystem studied but to other institutions in similar settings.

## Supporting Information

S1 Appendix Biomedical Waste (Management and Handling) Rules 1998, Ministry of Environment and Forest, Government of India. (DOC)

S2 Appendix Biomedical Waste (Management and Handling) Rules 2011, Ministry of Environment and Forest, Government of India. (DOCX)
