# Supplementary material for: Staff Perception on Biomedical or Health Care Waste Management: A Qualitative Study in a Rural Tertiary Care Hospital in India
Source: PLoS One. 2015 May 29;10(5):e0128383. doi: 10.1371/journal.pone.0128383 (PMC4449010; doi:10.1371/journal.pone.0128383)
Supplement: S1 Appendix — (DOC) [file pone.0128383.s001.doc]

### SCHEDULE I

(See Rule 5)

### CATEGORIES OF BIO-MEDICAL WASTE

| Option | Waste Category | Treatment & Disposal |
| --- | --- | --- |
| Category No. I | Human Anatomical Waste  (human tissues, organs, body parts) | Incineration @/deep burial* |
| Category No. 2 | **Animal Waste**  (animal tissues, organs, body parts carcasses, bleeding parts, fluid, blood and experimental animals used in research, waste generated  by veterinary hospitals colleges, discharge from hospitals, animal) houses) | Incineration @ / deep burial* |
| Category No 3 | **Microbiology & Biotechnology Waste**  (wastes from laboratory cultures, stocks or specimens of micro-organisms live or attenuated vaccines, human and animal cell culture used in research and infectious agents from research and industrial laboratories, wastes from production of biologicals, toxins, dishes and devices used for transfer of cultures) | local autoclaving / micro-waving / incineration@ |
| Category No 4 | **Waste sharps**  (needles, syringes, scalpels, blades, glass, etc. that may cause puncture and cuts. This includes both used and unused sharps) | disinfection (chemical treatment @ 01/auto claving / micro- waving and mutilation/ shredding" |
| Category No 5 | **Discarded Medicines and Cytotoxic drugs**  (wastes comprising of outdated, contaminated and discarded medicines) | Incineration @/destruct ion and drugs disposal in secured landfills drugs disposal in secured |
| Category No 6 | **Solid Waste**  (Items contaminated with blood, and body fluids including cotton dressings, soiled plaster casts, lines, beddings, other material  contaminated with blood) | Incineration @ autoclaving / micro-waving |
| Category No. 7 | **Solid Waste**  (wastes generated from disposable items other than the waste sharps such as tubings, catheters, intravenous sets etc). | disinfection by chemical  treatment @ @ autoclaving/micro-waving and mutilation/ shredding## |
| Category No. 8 | **Liquid Waste**  (waste generated from laboratory and washing, cleaning, house-  keeping and disinfecting activities) | disinfection by chemical treatment@@ and discharge into drains. |
| Category No. 9 | **Incineration Ash**  (ash from incineration of any bio-medical waste) | disposal in municipal landfill |
| Category No. 10 | Chemical Waste  (chemicals used in production of biologicals, chemicals used in disinfection, as insecticides, etc.) | chemical treatment @@ and discharge into drains for liquids and secured landfill for solids |

@@ Chemicals treatment using at least 1% hypochlorite solution or any other equivalent chemical reagent. It must be ensured that chemical treatment ensures disinfection.

## Multilation/shredding must be such so as to prevent unauthorised reuse.

@ There will be no chemical pretreatment before incineration. Chlorinated plastics shall not be incinerated.

- Deep burial shall be an option available only in towns with population less than five lakhs and in rural areas.

+ Options given above are based on available technologies. Occupier/operator wishing to use other State-of-the-art technologies shall approach the Central Pollution Control Board to get the standards laid down to enable the prescribed authority to consider grant of authorization.

**SCHEDULE II**

(see Rule 6)

### COLOUR CODING AND TYPE OF CONTAINER FOR DISPOSAL OF BIO-MEDICAL WASTES

| **Colour Coding** | **Type of Container -I** | **Waste Category** | **Treatment options as per Schedule I** |
| --- | --- | --- | --- |
| Yellow | Plastic bag | Cat. 1, Cat. 2, and Cat. 3,  Cat. 6. | Incineration/deep burial |
| Red | Disinfected container/plastic bag | Cat. 3, Cat. 6, Cat.7. | Autoclaving/Microwaving/  Chemical Treatment |
| Blue/White  translucent | Plastic bag/puncture proof Container | Cat. 4, Cat. 7. | Autoclaving/Microwaving/  Chemical Treatment and  destruction/shredding |
| Black | Plastic bag | Cat. 5 and Cat. 9 and  Cat. 10. (solid) | Disposal in secured landfill |

**Notes:**

1. Colour coding of waste categories with multiple treatment options as defined in Schedule I, shall be selected depending on treatment option chosen, which shall be as specified in Schedule I.

2. Waste collection bags for waste types needing incineration shall not be made of chlorinated plastics.

3. Categories 8 and 10 (liquid) do not require containers/bags.

4. Category 3 if disinfected locally need not be put in containers/bags.
