# Supplementary material for: Staff Perception on Biomedical or Health Care Waste Management: A Qualitative Study in a Rural Tertiary Care Hospital in India
Source: PLoS One. 2015 May 29;10(5):e0128383. doi: 10.1371/journal.pone.0128383 (PMC4449010; doi:10.1371/journal.pone.0128383)
Supplement: S2 Appendix — (DOCX) [file pone.0128383.s002.docx]

**SCHEDULE –I**

**(See rule 4 and 7)**

**CATEGOROES OF BIO-MEDICAL WASTE**

| **Category** | **Waste category(Type)** | **Treatment and disposal Option** |
| --- | --- | --- |
| CategoryNo.1 | **Human Anatomical waste**  (human tissues ,organs, body parts) | Incineration@@ |
| CategoryNo.2 | **Animal waste**  (animal tissues ,organs, body parts carcasses, bleeding parts, fluid, blood and experimental animal used in research ,waste generated by veterinary hospitals/colleges ,discharge from hospitals, animal house) | Incineration@@ |
| CategoryNo.3 | **Microbiology & Biotechnology waste and other laboratory waste**  (waste from clinical samples, pathology, bio-chemistry ,hematology, blood bank, laboratory cultures, stocks or specimens of micro-organism live or attenuated vaccines ,human and animal culture used in research and industrial laboratories, wastes from production of biological, toxins, dishes and devices used for transfer of cultures) | Disinfection at source by chemical treatment@ or by autoclaving/microwaving followed by multilation/shredding## and after treatment final disposal in secured landfill or disposal of recyclable wastes (plastics or gases) through registered or authorized recyclers.) |
| CategoryNo.4 | **Waste sharps**  (Needles, glass. Syringes or Syringe with fixed needles, scalpels, blades, glass etc. that may cause puncture and cuts. This includes both used and unused sharps ) | Disinfection by chemical treatment@ or destruction by needles and tips cutters, autoclaving/microwaving followed by mutilation/shredding## , whichever is applicable and final disposal though authorized CBWTF or disposal in secured landfill or designated concrete waste sharp pit. |
| CategoryNo.5 | **Discarded Medicines and Cytotoxic drugs**  (waste comprising of outdated, contaminated and discarded medicines) | Disposal in secured landfill or Incineration@@ |
| CategoryNo.6 | **Soiled waste**  (Items contaminated with blood ,and body fluids including cotton ,dressings ,soiled plasters casts, linen, beddings, other material contaminated with blood) | Incineration@@ |
| CategoryNo.7 | **Infectious solid waste**  (Wastes generated from disposal items other than the waste sharps such as tubings , hand gloves ,saline bottles with IV tubes, catheters ,glass, intravenous sets etc.) | Disinfection by chemical treatment@ autoclaving/microwaving followed by mutilation/shredding## and final disposal through registered or authorized recyclers |
| CategoryNo.8 | **Chemical waste**  (Chemicals used in production of biological ,chemicals used in disinfection ,as insecticides etc.) | Chemical treatment and discharge into drain meeting the norms notified under these rules and solids disposal in secured landfill. |

Notes:

@ *Chemicals treatment using at least 1% hypochlorite solution or any other equivalent chemical reagent. It must be ensured that chemical treatment ensures disinfections*

*## Mutilation/shredding must be such that so as to prevent unauthorized reuse.*

*@@ There will be no chemical pretreatment before incineration .Chlorinated plastics/bags shall not be incinerated.*

**SCHEDULE –II**

**(See rule 8)**

**COLOUR CODING AND TYPE OF CONTAINER FOR DISPOSAL**

**OF BIO MEDICAL WASTES**

| **Colour coding** | **Type of container to be used** | **Waste category Number** | **Treatment options as per schedule I** |
| --- | --- | --- | --- |
| Yellow | Non- chlorinated plastic bags | Category 1,2,5,6 | Incineration |
| Red | Non- chlorinated plastic bags /Punctured proof containers for sharps | Category 3,4,7  (4-waste sharps)  (In the earlier Rules, Soiled wastes are for Red colour) | As per schedule I  (rule 7) |
| Blue | Non- chlorinated plastic bags container | Category 8  (Chemical waste) | As per schedule I  (rule 7) |
| Black | Non- chlorinated plastic bags | Municipal Waste | Disposal in Municipal dump sites |

*Notes:*

1. Waste collection bags for waste types needing incineration shall not be made of chlorinated plastics

.

1. Category 3 if disinfected locally need not be put in containers / non-chlorinated plastic bags.
2. The municipal waste such as office waste (like paper waste),kitchen waste , food waste and other non infectious waste shall be stored in black coloured containers /bags and shall be disposed of in accordance with Municipal Solid Waste (Management and Handling)Rules2000.
